# Pain and Vertebral Dysfunction in Dry Immersion: A Model of Microgravity Simulation Different from Bed Rest Studies

**DOI:** 10.1155/2017/9602131

**Published:** 2017-07-13

**Authors:** L. Treffel, N. Massabuau, K. Zuj, M.-A. Custaud, G. Gauquelin-Koch, S. Blanc, C. Gharib, C. Millet

**Affiliations:** ^1^Université de Strasbourg, Strasbourg, France; ^2^Institut Pluridisciplinaire Hubert Curien, CNRS UMR 7178, Strasbourg, France; ^3^Université Claude Bernard Lyon 1, Lyon, France; ^4^Centre de Recherche Clinique, Centre Hospitalo-Universitaire d'Angers, CNRS UMR 6214, Angers, France; ^5^Centre National d'Etudes Spatiales (CNES), Paris, France; ^6^Service d'Odontologie, Hospices Civils de Lyon, Lyon, France

## Abstract

**Background:**

Astronauts frequently experience back pain during and after spaceflight. The aim of this study was to utilize clinical methods to identify potential vertebral somatic dysfunction (VD) in subjects exposed to dry immersion (DI), a model of microgravity simulation.

**Method:**

The experiment was performed in a space research clinic, respecting all the ethical rules, with subjects completing three days of dry immersion (*n* = 11). Assessments of VD, spine height, and back pain were made before and after simulated microgravity.

**Results:**

Back pain was present in DI with great global discomfort during the entire protocol. A low positive correlation was found (Pearson *r* = 0.44; *P* < 0.001) between VD before DI and pain developed in the DI experiment.

**Conclusions:**

There is a specific location of pain in both models of simulation. Our analysis leads to relativizing constraints on musculoskeletal system in function of simulation models. This study was the first to examine manual palpation of the spine in a space experience. Additionally, osteopathic view may be used to select those individuals who have less risk of developing back pain.

## 1. Introduction

Since space studies are limited, various ground-based analogs have been developed to simulate human existence in spaceflight. These methods include bed rest, head-out water immersion, and head-out dry immersion [1]. Among these, bed rest is the most commonly used method even if it does not eliminate the Gx (transverse G-Stress) gravitational influence. Removal of Gx stimuli can only be achieved in space or possibly reduced in the dry immersion model. Dry immersion (DI) involves immersing the human body in water while being covered with waterproof cloth to keep the subject dry. Whole body physiological changes have been observed after exposure to real or simulated microgravity [2]. Exposure to microgravity eliminates gravitational loads to the spine and, therefore, results in vertebral deconditioning which is characterized by a lengthening of the spine, muscular atrophy, back pain, and herniated discs [3–5]. In addition to muscle atrophy [5], height has been found to increase by 4 to 6 cm after spaceflight [6] and approximately 2 cm after simulated microgravity by head-down bed rest (HDBR) [3]. Kerstman et al. (2012) [7] reported that 52% of astronauts experienced back pain during spaceflight, which may be related to the development of herniated discs. Upon return to Earth, 10.0% (32 of 321) of US astronauts were diagnosed with intervertebral disc (IVD) herniation in comparison to only 3.5% (34 of a total of 983) of the Earth-based, control population [5, 8]. Of the cases reported, disc herniation is 21 times more likely in the cervical region and 3 times more frequent in the lumbar region. Due to its prevalence, the study of vertebral deconditioning has been recommended by the European Space Agency to determine and understand the underlying mechanisms of IVD herniation after spaceflight and to design countermeasures able to prevent or reduce vertebral deconditioning [5].

The pathophysiology of microgravity induced vertebral deconditioning has been addressed in the literature and it is likely to be discogenic and somatic [5, 7]. The lack of gravity forces can induce the impairment or abnormality in several components of spinal stability including the facet joints, intervertebral discs, ligamentous tissues, paraspinal muscles, and the nervous system [9–11]. Consequently, the damage of these structures maybe correlates to the lumbar back pain in astronauts. Therefore, information regarding vertebral deconditioning during simulated microgravity may help elucidate the etiology of back pain during spaceflight.

The global deconditioning with spaceflight affects all systems, particularly metabolic, cardiovascular, muscular, and vestibular systems. Concerning vertebral deconditioning defined by a spine lengthening, muscular atrophy, and back pain, there is no sufficient preventive approach except exercise countermeasures [4, 12, 13] and nutritional countermeasures [14, 15]. For example, physical resistive exercise with vibration, applied in the international space station and simulation studies, is commonly used in order to simulate loading on bone skeleton like on Earth in 1*G* [16, 17]. As countermeasures for back pain are limited, we propose that a new complementary and functional approach to back pain assessment by using vertebral manual testing, with an osteopathic approach, would be beneficial to optimizing subject selection before spaceflight and would assist in development of individualized countermeasures.

To the best of our knowledge, manual tests have never been performed during real or simulated spaceflight studies. Classic vertebral manual tests are used in clinical settings in osteopathy to investigate spine mobility in flexion, extension, and lateral motions. For these tests, a trained clinician palpates the spine to seek out vertebral somatic dysfunction (VD) through passive movements of the vertebra. It is known that inactivity associated with microgravity exposure contributes to deconditioning after spaceflight [18]. Therefore, manual testing of vertebral mobility is a noninvasive methodology that could be used to identify and evaluate vertebral zones of restricted movement before and after microgravity exposure. Manual approach presents the advantage of being painless and preventive with a functional regard on pain development. An osteopathic approach has never been used in real or simulated spaceflight studies. Our hypothesis is that manual testing could anticipate back pain developed in simulated or real microgravity. The objective of this study was to analyze the constraints in simulated microgravity by DI to better understand back pain development. The DI model is less commonly used than continuous −6° HDBR but is considered to be a more rapid model to simulate the effects of microgravity [19, 20]. Even if duration is only 3 days, significant results on musculoskeletal system, like muscle atrophy and postural disturbances, have been observed [20, 21]. A significant decrease in rectus femoris tone of over 10% has been seen and, at the cellular level, 3 days of DI leaded to a significant atrophy of type I muscle fibers [21].

## 2. Materials and Methods

### 2.1. Participants

**Figure 1:**
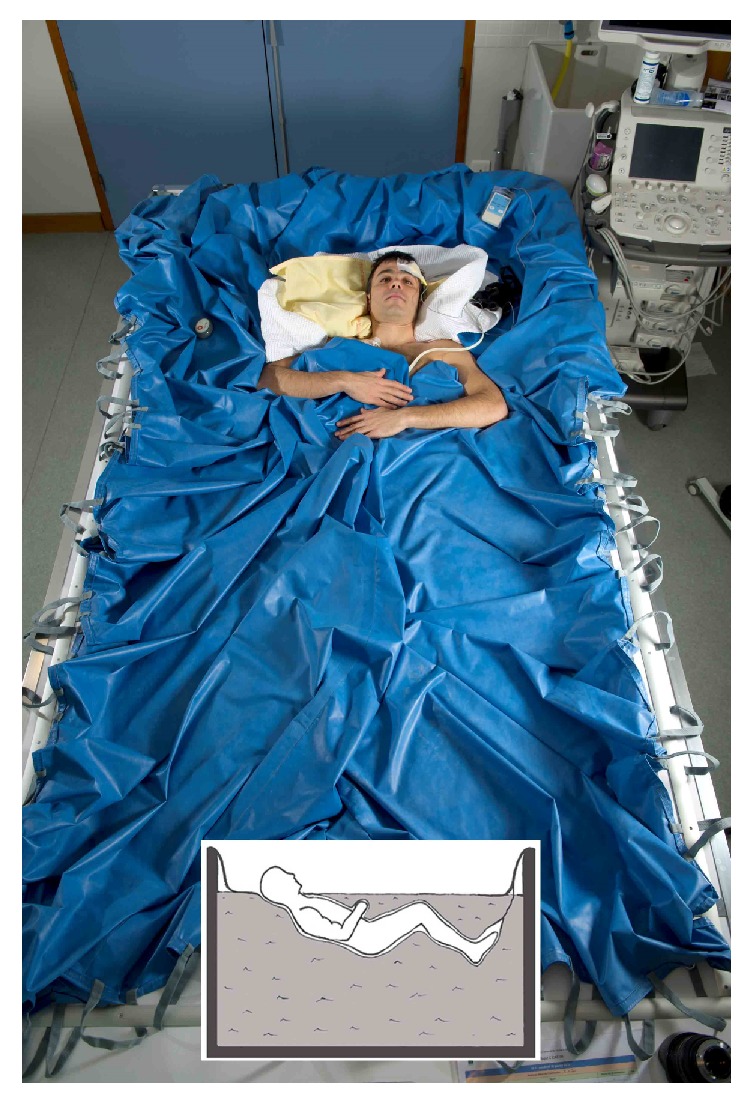
Dry immersion model in MEDES-IMPS Toulouse, France (Source CNES, 2015).

Eleven healthy male subjects (age: 31.8 ± 4.8 yr; height: 178.8 ± 6.7 cm; weight: 74.8 ± 7.0 kg; body mass index: 23.6 ± 1.5; aerobic fitness: 38.8 ± 2.9 ml·kg^−1^·min^−1^, Mean ± SD, one day before DI) were selected for the study (3 days in DI, Figure 1). Participants were selected based on a detailed medical history, physical examination, electrocardiogram, general blood screening, and urine analyses. In particular all participants were nonsmokers, were not using medication or other drugs, and were free from muscular and neurological pathologies. Study design was established in accordance with the Declaration of Helsinki and was approved by the local ethics committee (CPP Sud-Ouest Outre-Mer I, France) as well as the French Health Authorities with the following references: Number ID RCB: 2014-A00904-43, CPP: 1-14-26, and ANSM: 140997 B-81 for the DI study. All participants gave written informed consent before participation.

### 2.2. Experimental Design

**Figure 2:**
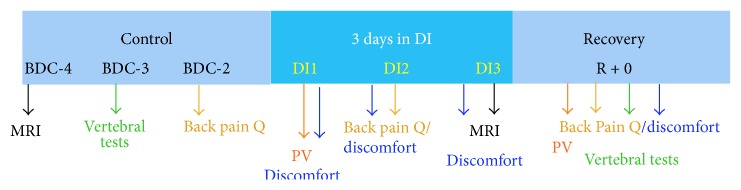
Tests resumed in chronology during all the experience. Tests used before dry immersion (control period: BDC for Base Data Collection), during the 3 days in DI (DI1, DI2, and DI3), and at recovery (MRI: Magnetic Resonance Imaging; back pain Q for Questionnaire, PV for Plasma Volume).

This experiment consisted of a 4-day ambulatory control period (BDC-4 to BDC-1), 3 days of dry immersion (DI 1 to DI 3), and 1 day of recovery (R + 0) (Figures 1 and 2). In the control and recovery periods preceding and following DI, all subjects remained active and ambulatory. All were asked not to exercise during the 8 days of the experiment. During DI, the subjects remained immersed in a supine position (Figure 1) in a controlled thermoneutral bath (33 ± 0.5°C) with the exception of the head and neck, which were not entirely immersed in water. During the study, eight different research groups performed their protocols on several physiological systems. These different protocols as well as daily weighting and toilet procedures involved water extraction. The total period out of immersion was 285 min for each subject. During this period the subjects were maintained in a −6° HDBR position.

The study was conducted in a quiet room at a temperature of ~25°C. Room lighting was on between 7:00 AM and 11:00 PM. Subjects received three solid meals/day during the experiment with the requirement to finish all meals. The individual energy intake was calculated by multiplying resting metabolic rate with a physical activity level of 1.6 during pre- and post-DI and 1.3 during DI. Only paracetamol was allowed if needed. Coffee, tea, alcohol, smoking, and drugs were prohibited throughout the experiment. The subjects were supervised by medical control and monitored 24 h per day. Each subject had a daily medical examination and MEDES personnel took several standardized measurements. Body temperature was taken twice daily with a tympanic thermometer. Heart rate and arterial blood pressure (systolic, mean, and diastolic) were measured every morning by means of an automated sphygmomanometer (Dinamap).

### 2.3. Experimental Procedure (Chronology of the Tests Resumed in Figure 2)

All the recovery data were measured less than one hour after the subject was first returned to an upright position (Recovery R + 0).

#### 2.3.1. Plasma Volume

The plasma volume was measured by CO-rebreathing method to verify if DI reproduces the same effects as those in HDBR or spaceflight. The plasma volume was also estimated in the morning (before breakfast) in supine position just before DI and immediately after DI just before standing (R + 0).

#### 2.3.2. Back Pain Questionnaire

A visual analog scale (VAS: 0–10 with 10 being the worst pain) was used by a medical doctor to determine the intensity and location of pain at BDC-2, at DI2, and at recovery (R + 0). Additionally, a global discomfort score (0–100 on VAS) was determined by questionnaire every morning and evening during the 3 days in DI. The question was “Do you feel global discomfort?” If yes, “what is the level of this discomfort from 0 to 100, where 0 is nothing and 100 the worst discomfort possible?”

#### 2.3.3. Spine Height

**Figure 3:**
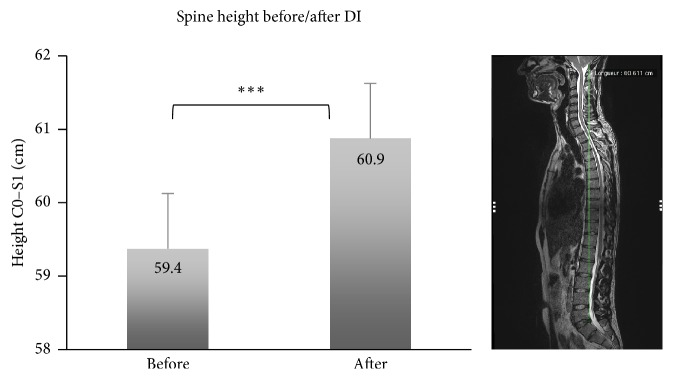
Comparison of spine height before and after dry immersion (DI) (Mean ± SD). Spine height between the foramen magnum (C0) and the superior and posterior endplate of the first sacral vertebra (S1). Comparison before and after DI (*P* < 0.001) (ANOVA repeated measures). *∗∗∗* means that *P* < 0.001.

Magnetic resonance imaging (MRI) was used to examine the spine in a supine position on day BDC-4 and the third day of DI (DI3). The spinal MR images were obtained using a scanner (MAGNETOM Avanto Syngo MR B17, TR: 1200 ms, TE: 119 ms; Siemens, Erlangen, Germany) with slice thicknesses of 1 mm. Subjects were kept in a −6° head-down position during the transfer to MRI clinic which took 10 to 15 minutes. The spine height was measured on sagittal T2 MR images between the foramen magnum of occipital bone (C0) and the posterior-superior endplate of the first sacral vertebra (S1) (Figure 3). The median sagittal plane crosschecking with the horizontal plane at the same level of the spine (superior endplate of C3) was used to measure with the same identical landmarks before and after DI. All of the MRI analysis was made with a Siemens engineer, using the OsiriX MD v.7.0.1 64-bit software.

#### 2.3.4. Vertebral Dysfunction

Vertebral manual tests were conducted before and after DI at BDC-3 and R + 0. Information about the subjects back pain was unknown to the professional osteopath during the tests to avoid potentially influencing the palpation. The classic osteopathic vertebral manual tests [22, 23] were used in order to identify vertebral somatic dysfunctions (VD). The research of VD is a convergence of clinical information (observation, characteristic of soft tissues, and test of mobility) to identify the dysfunctional zone. All the tests are passive and did not induce any pain. As described by Clem et al. [24], a VD is considered to be a restriction of movement. The osteopath investigator seeks out vertebral segments through mobility passive tests. Palpation plays a major role, particularly with regard to the identification of paraspinal soft tissue texture changes and altered intervertebral joint mobility, with altered tissue texture and joint mobility considered as the two most relevant clinical signs for the diagnosis of somatic dysfunction [25]. The maximum of 3 most blocked VD was noted per subject with the most evident clinical sign as restriction in vertebral segmental motion in flexion, rotation, and lateral bending. To note a vertebral level as in dysfunction the clinician tests the entire spine in all passive segmental motion. The ≤3 most relevant vertebrae were noted as VD.

#### 2.3.5. Hand-to-Ground Distance

**Figure 4:**
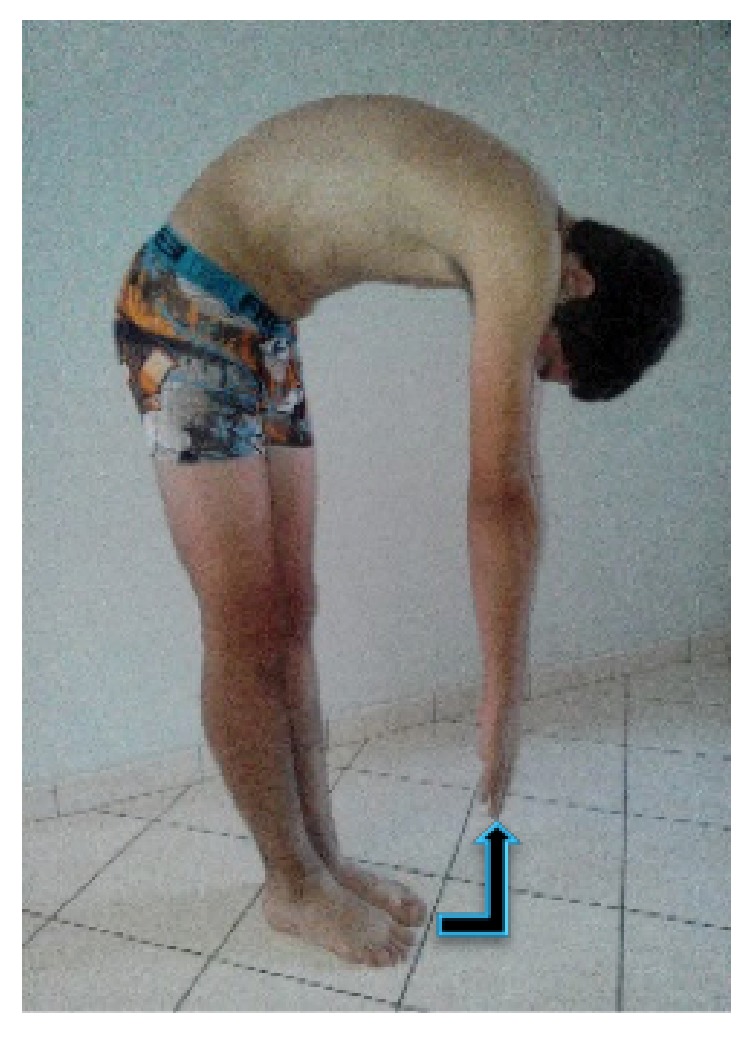
Hand-to-ground distance (in cm) tested before and after dry immersion. The movement is progressive and in sagittal plan without compensation. Legs are totally in extension with both feet tied. The goal is to show a restriction of spine mobility in flexion bending.

After the completion of manual tests, forward flexion of the spine was determined by measuring the distance between the ground and the patient's fingers (hand-to-ground distance (HTGD)) (Figure 4). The movement was progressive in the sagittal plane without compensation. Throughout the movement, legs were in full extension with both feet tied. This measure is commonly used in clinical practice and was used in the current study to determine the restriction of movement in flexion bending after DI.

### 2.4. Statistical Analysis

Statistical analyses were performed by Excel and StatPlus v5.9.80 software. The differences in means for each dependent variable were evaluated by repeated measures ANOVA with overall Fisher's protected least significant difference post hoc tests used to compare the means before and after both experimental conditions. Correlation between osteopathic tests and pain was examined by calculating the Pearson correlation coefficient (*r*). A significance level of *P* ≤ 0.05 was used for all tests. Results are presented as mean ± standard deviation.

## 3. Results

The plasma volume decreased significantly by 17%, from 3727.3 ± 109.9 mL at DI1 to 3095.6 ± 86.7 mL at recovery (*P* < 0.0001). A significant increase in height was observed in MRI after the experiment (Figure 3) from 59.4 ± 2.6 cm to 60.9 ± 2.7 cm (mean ± SD; *F*_(1,11)_ = 7.349, *P* < 0.001). During all the 3 days of DI, subjects described a global discomfort score of 40 ± 23/100 in average (0 is no discomfort, 100 is the worst discomfort) (Figure 6).

**Figure 5:**
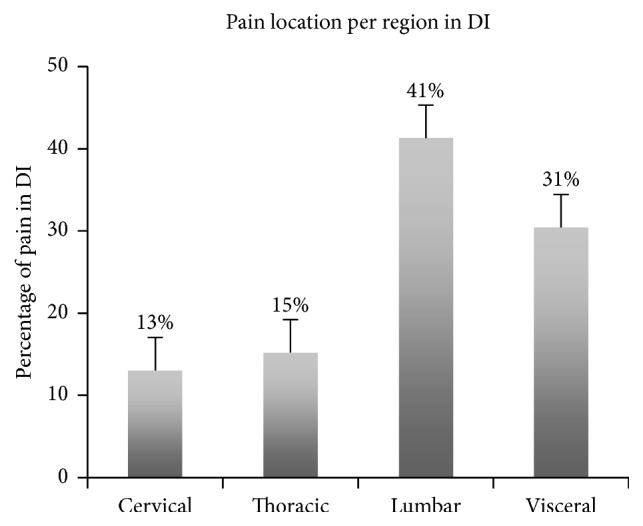
Pain location noted in percentage per region during a 3-day dry immersion (DI). Pain location in DI in percentage of apparition per region (% on 46 pains).

**Figure 6:**
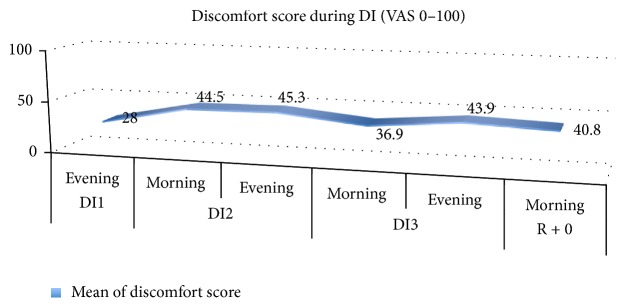
Discomfort during the 3 days in dry immersion (DI1, DI2, and DI3) and at recovery (R + 0). Evaluation by questionnaire using visual analog scale (0–100), with 100 being the most severe level.

Concerning the pain development, 92% of subjects (11/12) reported back pain during the 3 days in DI. Pain was primarily located in the lumbar (41%) and ventral/subcostal (31%) regions (Figure 5). Pain intensity was scored between 0 and 10 with 10 being the most severe level. The maximal of pain intensity reported during DI was on DI2 with a score of 3.75 ± 2.4/10 for the lumbar region. The intensity of lower back pain decreased significantly (3.75 to 1.75/10; *F*_(1,11)_ = 2.471; *P* = 0.02) during recovery (R0) from DI (R0: 1.75 ± 1.5/10) [26].

A significant increase in hand-to-ground distance (HTGD) was noted after a 3-day DI. The distance increased significantly +12.0 ± 5.2 cm (*F*_(1,11)_ = 8.08; *P* < 0.001) after DI [26]. This result suggests a restriction of spine mobility in flexion after microgravity exposure.

**Figure 7:**
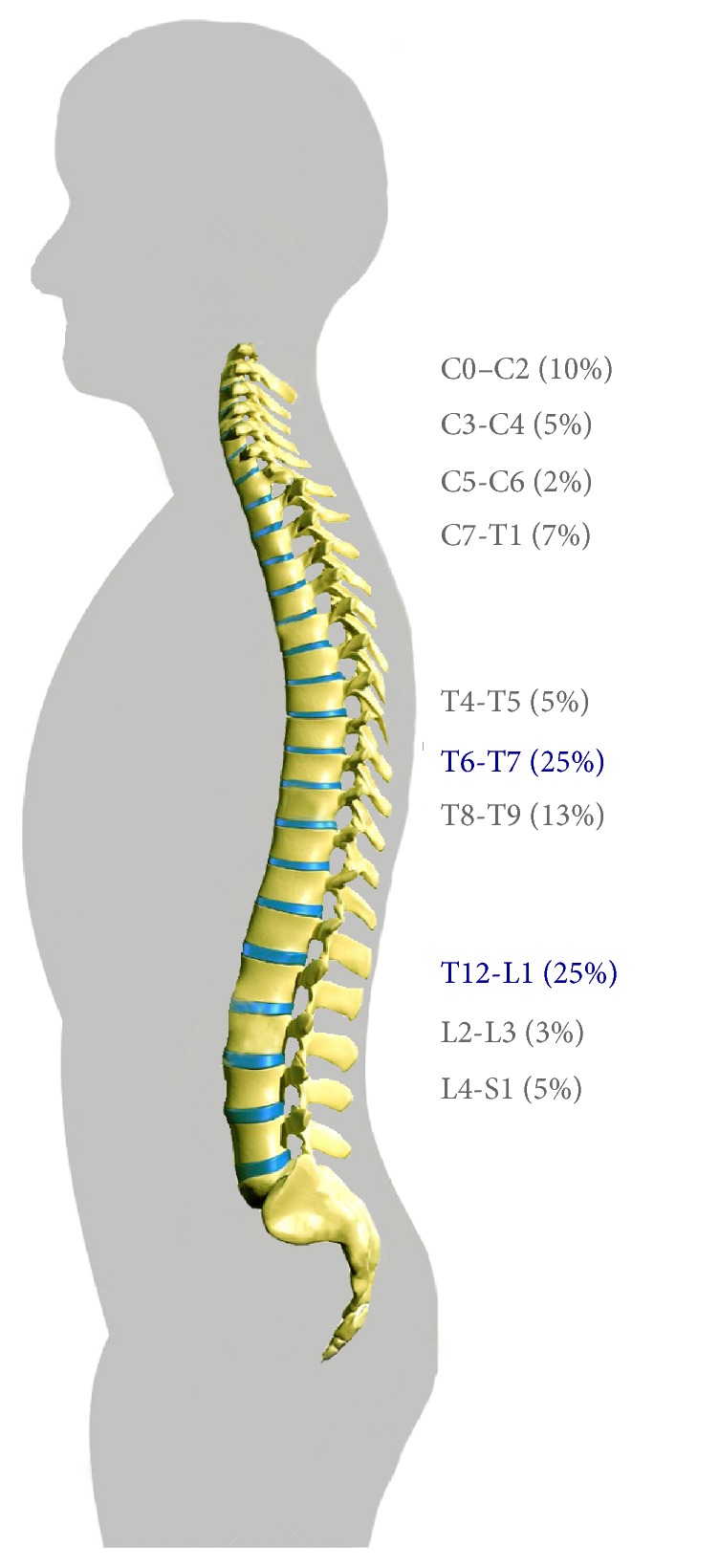
Repartition of vertebral somatic dysfunctions at recovery (R + 0) just after 3-day dry immersion (DI). The thoracolumbar (T12-L1) and thoracic (T6-T7) would be more frequently concerned after DI.

The spine presented vertebral dysfunction (VD) in different locations (Figure 7). VDs were particularly located in T12-L1 for 83% of subjects. A low positive correlation was found (Pearson *r* = 0.44;* T*-test *H*_0_: *P* < 0.001) between VD found before DI and pain developed in the DI experiment. This result could suggest a predictive value of VD as a risk factor of pain development.

## 4. Discussion

The aim of this study was to investigate pain and vertebral dysfunctions in humans during dry immersion, an analog model simulating microgravity. Although not a perfect simulation of spaceflight, particularly for the cervical region, the DI model can help to understand thoracic and lumbar vertebral deconditioning observed after spaceflight. To our knowledge, the present study was the first to examine the development of back pain with vertebral osteopathic tests in a simulation of microgravity exposure. The main results were (1) a high level of global discomfort during the 3 days in DI, (2) an increase in height and HTGD after experiment, and (3) vertebral dysfunctions predominately developed in the thoracolumbar region.

Although several factors may influence plasma volume, the decrease of 17% seen in the present study is comparable to that observed in space [19, 27]. Furthermore, results about blood changes in DI have been observed [26]. For example, Plasma Na+ and estimated plasma osmolarity increased from BDC-1 to DI2. All these values were in normal range. Many fundamental differences exist between spaceflight and ground-based simulation models, and between the different models [1]. In general, simulated microgravity studies do not include factors such as launch stress, acceleration, excitement, anxiety, prolonged isolation, and environmental parameters such as light intensity, light/dark cycles, pressure, and radiation exposure. For example, spaceflights are associated with increased stress levels, while DI and HDBR are associated with boredom due to monotony and immobilization [28]. So all or any of these factors constitute limitations, which could affect the interpretation of the results of simulation studies. Moreover, the microgravity environment is an important causal factor for spaceflight induced sensorimotor changes [29].

**Figure 8:**
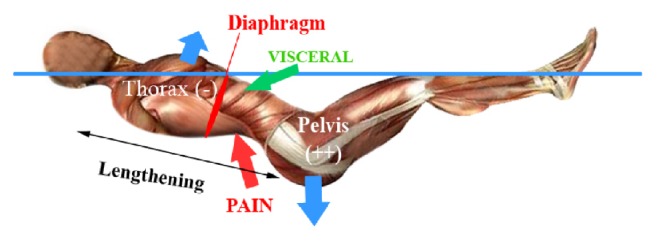
Explicative schematic representation of uncomfortable position in dry immersion (lengthening of spine, visceral mass under diaphragm muscle, and preferential pain in thoracolumbar region) (Published in Treffel et al., 2016 [26]).

Head-down bed rest (HDBR) is commonly used as a model for microgravity exposure. Although DI and HDBR are considered to be two models simulating microgravity, the models elicit different biomechanical responses potentially influencing the interpretation of the effects of spaceflight on vertebral somatic dysfunction. Forces applied on body would be specific in the DI model (Figure 8), which is different than in supine position like in HDBR. In contrast, DI may not be a good model to simulate microgravity effects on cervical region. As demonstrated by authors, intervertebral disc volume is not changed in cervical discs after DI experience [26].

As in spaceflight both exposure to HDBR and DI resulted in an increase in height. In DI, increase in spine length may translate into increases in total length of comparable magnitude to that observed in HDBR considering that part of the total length increase has to be ascribed to stretching of the lower limb joint capsules in addition to the increase in spine length [3].

The HTGD measurement provides an indication of mobility dependent on the movement capacity of the spine, the hip joint, and the posterior muscles. Therefore, this measure was conducted before and after DI and HDBR to determine the global restriction of movement, which is characteristic in the manual approach of assessing vertebral dysfunction. The results of the current study showed a significant restriction of movement post-DI similar to that observed in a HDBR study suggesting a stiffening of posterior myofascial chains resulting in ankylosis and a decrease in intramuscular pressures [30]. Inactivity during the studies may have contributed to this observed change. Previous work has reported muscular atrophy, particularly prevalent in the lower limb [31], mainly the deep posterior compartments [30]. This is expected as the lower limb muscles, which are normally used to maintain an upright posture [30] and for locomotion on Earth, are not as active during spaceflight and simulated microgravity. A cellular analysis of postural muscle would be interesting to examine the possibility of increased collagen contributing to reducing mobility after microgravity exposure [5]. Moreover, the reduction of the tonic drive of vestibulospinal volleys in microgravity could influence the muscle tropism. The link between VD and gait postural performance has yet to be proven. For Walser et al. (2009) an osteopathic treatment could influence positively the balance control [32]. Further studies have to investigate the underlying mechanisms of motion capacity after spaceflight in order to explain this ankylosis, clinically observed as vertebral motion restriction.

Subjects in both simulations of microgravity (DI and HDBR) developed back pain [33, 34], but with different characteristics. The cervical region was affected more during the HDBR (33%, personal data) than in DI (13%). Moreover, we observed in DI that there is a visceral pain (in 31% of subjects), which is not present in HDBR. DI model has been shown to reproduce the effects of microgravity on musculoskeletal system [19, 21] and postural consequences [20]; however, the body position is very specific and could explain the strains applied on body during DI (Figure 8). With DI there is an opposition of forces applied on spine resulting in the generation of pain. As the pelvis is heavy it has a tendency to sink during DI, which is in contrast to the thorax, which has a tendency to rise because it is full of air. This opposition of forces leads to an increased stress on the thoracolumbar region. Moreover, in DI the visceral mass is drawn towards the diaphragm muscle, which could explain the concentration of pain in the lumbar region and a visceral subcostal pain (31% of pain in DI, Figure 8). The lower limbs are in flexion like in space. We clinically observed the hips flexion, which leads to a retroversion of pelvis. This phenomenon could facilitate the decrease in lumbar curvature [26], and finally the similar spine lengthening observed in space. In our opinion we can make a relative parallel between the posture in flexion in DI and the posture in space, as described in Buckey [35]. The specific forces applied on spine in DI are very accurate to simulate the vertebral deconditioning observed after spaceflight and HDBR. It is particularly true for lumbar region in DI model. The position in HDBR and in DI seems to be different and could explain the differences in pain and VD location described by subjects in both models. This highlights that the development of back pain is a function of the model used despite similar changes in global deconditioning as that described after spaceflight.

**Figure 9:**
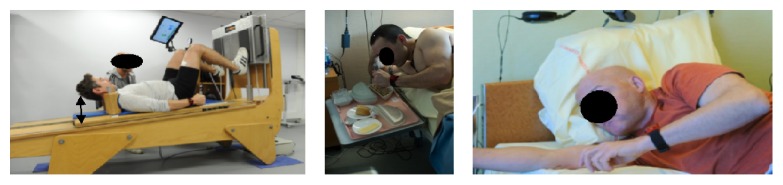
The posture used during head-down bed rest could explain cervical somatic dysfunction and muscle hypertrophy (Source: CNES, MEDES).

Exposure to microgravity is associated with both decreased amplitude and frequency of spine motion [36, 37], potentially resulting in the development of vertebral somatic dysfunction (VD) resulting in pain and global discomfort. Similar to the development of pain, both models of microgravity exposure resulted in the development of VD, but the different biomechanical stimulations of each resulted in different patterns of VD and pain. In HDBR (personal data) the majority of VD was located in crania-cervical (21% in C0–C2) and lumbar (16% in L2–L4) regions. This cervical location in bed rest could be explained by the decline position and a flexion of head to compensate for the −6° head-down position. For example, for meals the subjects used cervical muscles or a hand in order to keep the head in an easier position to ingest foods (Figure 9). This observation is in agreement with the results showed by Belavý et al. (2013) [38] that during HDBR there is a hypertrophy of the cervical muscles. With respect to the thoracic location of VD in T4-T5 and T8-T9 (personal data), it is possible that the upward shift in body mass with the head-down position contributed to increased force required during respiration resulting in strain in the thoracic region. Additionally, VD in the T5–T9 region is consistent with the anatomic nerve link with the greater thoracic splanchnic nerve [39], which could develop referred pain with nociceptive processing pathways for visceral and vertebral somatic inputs [40, 41].

In DI our results showed a different location of the vertebral somatic dysfunctions. The VDs most frequently identified were T6-T7 and T12-L1. The thoracolumbar dysfunction was understandable due to the specific position with the opposition of forces between thorax and pelvis experienced during DI. The T6-T7 location of VD could be linked to the visceral subdiaphragmatic pain (Figure 8). Particularly, almost all subjects (11/12) described pain on the left side seemingly linked to the stomach with radicular, left costochondral, and epigastric pain. In all cases, this visceral pain was increased when inhaling air, or, more precisely, when the diaphragm muscle was activated, increasing pressure in abdomen. This suggests that movement of the visceral mass during DI contributed to the development of vertebral somatic dysfunctions resulting in referred pain and prolonged global discomfort [41]. We would like to emphasize that the other experimentations (e.g., intraocular pressure or cardiovascular measurements with echography [42]) did not induce any bias in pain and discomfort estimation.

This pilot study using vertebral manual tests in simulation study aims at raising the interest of osteopathic vertebral analysis in models of microgravity exposure. The study was intended to highlight differences in the testing methods and exact interpretation of spinal mobility testing may be problematic. Indeed, many variables may affect accuracy and reliability of spinal motion palpation (tactile perception) used by osteopaths: fingertip utilized, intensity of the manual pressure, speed of the test motion induction, visual focus to the tactile task, attentiveness to the motion exam, fingertip skin conformation to the spatial details of the body surface being palpated, texture of the surface, age of the examiner, and frequency of use of the motion palpation [43, 44]. For example, a slow induction of spinal movement during testing may enhance tactile discrimination. The attention of examiner during spinal palpation may determine the outcome interpretation. However, investigation of the validity and reliability of spinal palpatory diagnostic tests has been in progress in recent years. Thereby, the reliability of the vertebral osteopathic tests is validated by scientific approach used in previous studies on back pain [45, 46]. Reproducibility of manual and more specifically segmental mobilization techniques remain a debatable matter in the literature. There seems to be a general tendency towards higher intraobserver reliability compared to interobserver results [45–48]. This is a new preventive approach of back pain, which has the advantage of being passive, noninvasive, and without any bias with other measurements. Manual analysis of subjects' spines before participating in a simulation study could be used with the aim to exclude potential subjects who would have a predisposing profile to developing back pain. Finally, we would like to add an osteopathic analysis before a microgravity exposure or a simulation study, and after with manipulative treatment in order to decrease back pain and herniated discs development [32, 49–51]. It seems to be important to identify and treat VD. Indeed, it has been observed that persistent vertebral motion restriction has an association with final lumbar bone mineral density *T* scores, and persistent tissue texture abnormalities and tenderness are associated with changes in the bone mineral density *T* scores [23]. Moreover, the osteopathic manipulative treatment could be used during real and simulated spaceflight with manual treatment being used as an alternative method to the ingestion of analgesic drugs. Further investigations are needed, with more osteopaths to limit interobserver bias.

## 5. Conclusions

The data add to the scientific literature on back pain in spaceflight simulations. This study demonstrated that osteopathic techniques can be used to assess back pain developed with DI. Additionally, back pain developed during DI was different from HDBR suggesting that vertebral deconditioning is dependent on the simulation model used. Our work represents a pilot study to assess the feasibility of vertebral manual analysis during spaceflight studies. The pain management during and after real or simulated spaceflight would have to include an osteopathic view, in order to identify the vertebral dysfunction for preventing back pain. The results showed an association of VD in preexposure conditions to pain developed during DI. Additionally, the results suggest that manual tests represent a tool that can be used to better understand and potentially decrease vertebral deconditioning experienced by astronauts, thereby leading to a new understanding of the pathophysiology of intervertebral disc problems experienced upon returning to Earth (as recommended by space agencies [5]).

Future work should be conducted to investigate the possibility of a vertebral manual treatment before or during the two models of microgravity exposure (HDBR and DI) as a countermeasure to decrease some effects of vertebral deconditioning. Thus, a manual therapy before and/or during experiments could decrease the ingestion of analgesic drugs and potentially improve the overall wellbeing of astronauts, patients, and study participants [52]. Moreover, a clinical and manual analysis of subjects' spine before participating in a simulation study could be used with the aim to exclude potential subjects who would have a predisposing profile to developing severe back pain. In our opinion the best pain management is to prevent pain development more than to treat it.

## Abbreviations

ANOVA: Analysis of variance
BDC: Base data collection
BP: Back pain
DI: Dry immersion
HDBR: Head-down bed rest
HTGD: Hand-to-ground distance
IVD: Intervertebral disc
R: Recovery
VAS: Visual analog scale
VD: Vertebral somatic dysfunction.
